# How Does a Carnivore Guild Utilise a Substantial but Unpredictable Anthropogenic Food Source? Scavenging on Hunter-Shot Ungulate Carcasses by Wild Dogs/Dingoes, Red Foxes and Feral Cats in South-Eastern Australia Revealed by Camera Traps

**DOI:** 10.1371/journal.pone.0097937

**Published:** 2014-06-11

**Authors:** David M. Forsyth, Luke Woodford, Paul D. Moloney, Jordan O. Hampton, Andrew P. Woolnough, Mark Tucker

**Affiliations:** 1 Arthur Rylah Institute for Environmental Research, Department of Environment and Primary Industries, Heidelberg, Victoria, Australia; 2 Ecotone Wildlife Veterinary Services, Canberra, Australian Capital Territory, Australia; 3 Invasive Plants and Animals Branch, Biosecurity Division, Department of Environment and Primary Industries, Melbourne, Victoria, Australia; 4 Melbourne Water, Reefton, Victoria, Australia; University of Alberta, Canada

## Abstract

There is much interest in understanding how anthropogenic food resources subsidise carnivore populations. Carcasses of hunter-shot ungulates are a potentially substantial food source for mammalian carnivores. The sambar deer (*Rusa unicolor*) is a large (≥150 kg) exotic ungulate that can be hunted throughout the year in south-eastern Australia, and hunters are not required to remove or bury carcasses. We investigated how wild dogs/dingoes and their hybrids (*Canis lupus familiaris*/*dingo*), red foxes (*Vulpes vulpes*) and feral cats (*Felis catus*) utilised sambar deer carcasses during the peak hunting seasons (i.e. winter and spring). We placed carcasses at 1-km intervals along each of six transects that extended 4-km into forest from farm boundaries. Visits to carcasses were monitored using camera traps, and the rate of change in edible biomass estimated at ∼14-day intervals. Wild dogs and foxes fed on 70% and 60% of 30 carcasses, respectively, but feral cats seldom (10%) fed on carcasses. Spatial and temporal patterns of visits to carcasses were consistent with the hypothesis that foxes avoid wild dogs. Wild dog activity peaked at carcasses 2 and 3 km from farms, a likely legacy of wild dog control, whereas fox activity peaked at carcasses 0 and 4 km from farms. Wild dog activity peaked at dawn and dusk, whereas nearly all fox activity occurred after dusk and before dawn. Neither wild dogs nor foxes remained at carcasses for long periods and the amount of feeding activity by either species was a less important predictor of the loss of edible biomass than season. Reasons for the low impacts of wild dogs and foxes on sambar deer carcass biomass include the spatially and temporally unpredictable distribution of carcasses in the landscape, the rapid rate of edible biomass decomposition in warm periods, low wild dog densities and the availability of alternative food resources.

## Introduction

Mammalian carnivores can have important ecological, economic and social impacts. Key ecological impacts of carnivores include the regulation of prey species and the suppression of smaller carnivores (‘mesopredators’), both of which may influence ecosystem structure and function [1]–[4]. Where carnivores and humans overlap, carnivores may kill humans and humans may kill carnivores [4], [5]. Another impact of carnivores on humans is predation on livestock reducing the economic viability of farming enterprises [6]. The actual and perceived impacts of carnivores on human activities can have undesirable social consequences [7]. There is therefore much interest in understanding how carnivores utilise food resources [8], and particularly those food resources that are provided directly or indirectly by people (i.e. anthropogenic [9]–[12]).

Many mammalian carnivores scavenge the carcasses of domestic livestock [7], [9], [13]. In Alberta, cattle carcasses at ranchers’ boneyards (where livestock are dumped) were an important food source for wolves (*Canis lupis*) during winter [9]. In Poland, the tracks of red foxes (*Vulpes vulpes*) were more likely to be observed around boneyards compared to other locations [14]. Another anthropogenic source of carcasses that could potentially be used by carnivores are ungulates shot by hunters. Ungulates, often the primary prey of apex carnivores (e.g. wolves [15] and African lion (*Panthera leo*) [4]), are increasing in North America [16], Europe [17] and Australia [18]. Ungulates are frequently subject to intensive harvesting by hunters [19]. If ungulate carcasses are not removed by hunters, then a substantial food source is available to be scavenged [20], [21].

Multiple carnivore species are often present in ecosystems, and there is potential for interspecific competition (including predation) at large food resources such as ungulate carcasses [22]–[24]. The larger and more aggressive species in the carnivore guild may be able to control access to carcasses [25], with subordinate species avoiding carcasses when dominant species are present [23], [24], [26]. Mutualistic and commensal interactions are also possible, for example if a large carnivore opens up an ungulate carcass then smaller carnivores may also be able to feed on the carcass [22], [23]. Hence, a hierarchy of access to carcasses is expected within carnivore guilds [27], [28].

Active management of large carnivores, usually by lethal control [7], is often undertaken to reduce their negative impacts. These removal activities could increase the availability of anthropogenic food resources such as carcasses to subordinate species. If the impacts of the subordinate carnivore are also undesirable, as is the case for many introduced mesopredators [3], then the combination of hunter-killed carcasses and the selective removal of the dominant predator through lethal control could lead to unexpected and undesirable ecological outcomes [29], [30].

Wild dogs (10−25 kg), comprising dingoes (*Canis lupus dingo*), feral dogs (*C. l. familiaris*) and hybrids of the two are present in much of mainland Australia [7]. Dingoes have been present in mainland Australia for at least 4000 years [31], but feral dogs have established following European contact (i.e. much more recently [7]). There is evidence that dingoes can reduce the abundance of macropod prey [32]–[35], with flow-on effects on vegetation structure and composition [33], [35]. Wild dogs injure and kill domestic livestock, causing economic and social distress to farming communities, and hence are actively managed in much of south-eastern Australia [7]. In the state of Victoria, wild dogs are subject to lethal control on private land and on public land within 3 km of private land [36]. Exotic red foxes (5−8 kg) and feral cats (*Felis catus*; 3−6 kg) have been implicated in the extinction of numerous small native marsupials [37], [38]. Foxes can kill up to 30% of lambs on individual farms [13], [39]. Whereas wild dogs and foxes commonly consume carrion [7], [13], [40], feral cats are thought to prefer live prey [37]. There is a large overlap in the diets of wild dogs and foxes in south-eastern Australia [41], and wild dogs can kill foxes and feral cats [42]. It has been suggested that the abundances (and hence impacts) of foxes and feral cats are greater where wild dogs have been controlled due to release from wild dog predation and competition [3], [38]. There is evidence from scat and sandplot studies that foxes and feral cats avoid locations used by wild dogs [41], . If there is interference competition and/or intraguild predation at carcasses, then we expect negative spatial and temporal relationships between dingoes and foxes, and between foxes and feral cats [43], [46].

**Figure 1:**
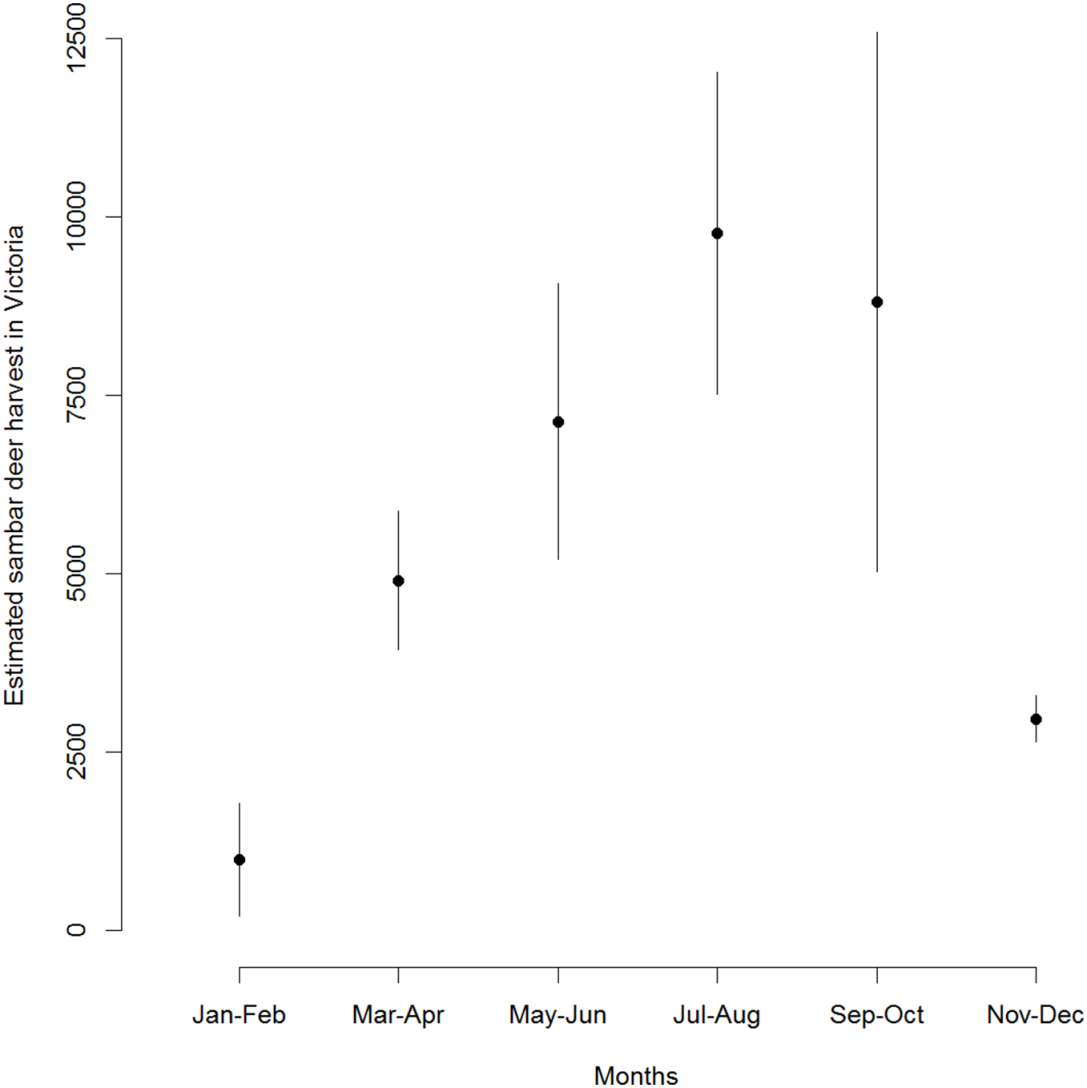
Mean (± SE) estimated bi-monthly harvests of sambar deer in Victoria, south-eastern Australia, 2009−2013. Data are summarised from references 54−58.

The sambar deer (*Rusa unicolor*, previously *Cervus unicolor* [47]) is the largest (males up to 250 kg and females up to 150 kg [48]) and most widespread deer species in Australia [49]. Native to India, Sri Lanka and south-eastern Asia [47], sambar deer were introduced into south-eastern Australia during the 1860s and have subsequently colonised large parts of Victoria and New South Wales [48], . The highest densities of sambar deer are in low-elevation areas where forest abuts productive grassland [53]. Hunting of sambar deer is a popular activity, with an estimated 32,000 harvested annually within Victoria alone [54]–[58]. Hunting deer requires a licence but there is no limit on the number or sex-age classes of sambar deer that can be harvested, and there are no regulations governing the disposal of carcasses (i.e. they do not have to be buried or otherwise removed) [59]. Most sambar deer are harvested during the months of May−October (i.e. the austral winter and spring; Fig. 1), likely increasing the availability of carcasses to scavengers due to reduced activity of insect and microbial decomposers compared to the warmer summer months [60]. Given the large number of sambar deer harvested annually, there is concern that their carcasses are an important food source for wild dogs and foxes.

In this study, we used remote cameras (‘camera traps’ [61]) to quantify spatial and temporal patterns of utilisation of hunter-shot deer carcasses by wild dogs, foxes and feral cats in south-eastern Australia. Since wild dogs are likely to have priority (i.e. dominance) access to carcasses over foxes and feral cats, control of wild dogs was expected to result in reduced activity of wild dogs and increased activity of foxes at deer carcasses at the farm-forest interface (i.e. spatial partitioning). We predicted that there would be temporal partitioning of feeding at carcasses, with foxes avoiding wild dogs and feral cats avoiding foxes. We also predicted that carcasses would remain available to carnivores for longer in the cooler winter months compared to the warmer spring months due to slower decomposition by insects and microbes in the former relative to the latter, but that the feeding activities of mammalian carnivores would hasten their decomposition.

## Materials and Methods

### Sambar Deer Carcasses

We obtained 30 sambar deer carcasses that were shot during a cull in the Upper Yarra Ranges National Park (37°43′S, 146°00′E), c. 100 km from Melbourne, south-eastern Australia. For more detail on sambar deer in this area see Forsyth et al. [53]. We eviscerated and halved all carcasses (head, front legs and ribs forming the front half and the remainder the rear half) to facilitate transport and freezing. Antlers were always removed. The stomach was not used in our study because of concerns about the possible transport of seeds [62] into our study area. As hunters typically open deer carcasses and remove body parts [28], our carcasses likely reflected the state of many carcasses in the south-east Australian landscape after being processed by hunters.

### Carcasses in the Landscape

**Figure 2:**
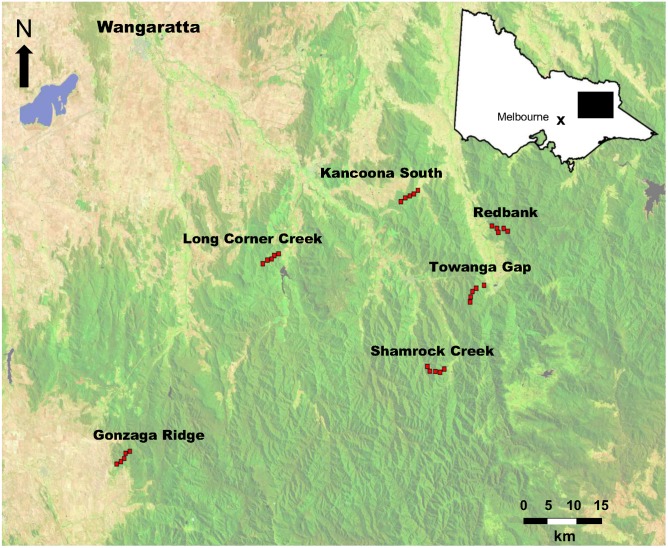
Location of the study in the North East region of Victoria, south-eastern Australia. Red squares indicate the location of sambar deer carcasses along the six transects. The ‘×’ in the inset indicates the location of the Upper Yarra Ranges National Park, where carcasses were obtained.

The 30 carcasses were placed along six transects in the North East region of Victoria, c. 250 km from Melbourne (midpoint of transects: 36°42′S, 146°58′N; Fig. 2). The landscape consists of valley bottoms that have been cleared of forest and are now sheep and cattle farms surrounded by State Forest. Wild dogs/dingoes, foxes, feral cats and sambar deer are present throughout the area [51], [52], [63]. Fallow deer (*Dama dama*) are patchily distributed in the area (M. Beach, Department of Environment and Primary Industries, personal communication). The North East region is popular for deer hunting [54]–[58], with no limit on the number of sambar deer or fallow deer that can be harvested on private land or State Forest [59]. State Government wild dog controllers routinely control wild dogs on farms and adjacent forest but do not conduct control more than 3 km into State Forest from private land. Most livestock farmers also control wild dogs on their farms (M. Beach, Department of Environment and Primary Industries, personal communication). Transect locations were selected in consultation with wild dog controllers and needed to meet four criteria: (i) wild dogs present in the area (foxes and feral cats were considered ubiquitous but, due to control, wild dogs may not be present) but not scheduled for wild dog management within the next six months; (ii) be public land containing sambar deer that can legally be hunted; (iii) abut livestock farms, and; (iv) be accessible by vehicle.

We placed deer carcasses at 1-km intervals along each transect, with transects starting c. 50 m inside the forest abutting farms and extending 4-km into forest on public land (Fig. 2). Hence, the distance of each carcass to a farm was as follows: carcass 1 = 0.05 km; carcass 2 = 1 km; carcass 3 = 2 km; carcass 4 = 3 km; and carcass 5 = 4 km. At the pre-determined straight-line distance from the farm, the vehicle was stopped and the two halves of the carcass were unloaded and dragged to the flattest location within 10–50 m of the vehicle track. A minimum distance of 10 m was chosen to minimize the probability of cameras being stolen and a maximum distance of 50 m was chosen because of the difficulties of dragging carcasses through forest. After clearing the site of understory vegetation, the front and rear carcass halves were tied to a star picket to prevent the carcass being dragged away. Two cameras [RECONYX HC600 Hyperfire H.O. Covert IR (RECONYX, Inc., Holmen, Wisconsin, USA)] were attached to trees at each carcass site such that they provided complementary views of the carcass. One camera was set to take photographs with no time delay and the other was set at 15 second intervals in order to prolong the survey period should the other camera run out of battery power or fill its SD card. Both cameras were set to high sensitivity for the entire 24-hour period and were programmed to record the date and time. The camera SD cards and batteries were replaced at approximately 14-day intervals.

Carcasses were placed along three transects in May 2012 and 2013 (‘winter’) and three transects in August−October 2012 (‘spring’). The elevation range of carcasses was 322−1046 m above sea level.

The edible biomass (i.e. excluding the skeleton, skin and hair [64]) of both carcass halves were estimated to the nearest 5% at each visit (i.e. at 14-day intervals) by the two senior authors. The edible biomass was estimated by eye without touching or moving the carcass, and the two senior authors consulted so that they were confident that the estimates were accurate and repeatable. Hence, an intact carcass half had an edible biomass of 100%. Cameras were removed from a carcass when its edible biomass was ≤10%. We used the mean % edible biomass of the two halves in our analyses because of the potential for edible biomass to decline at varying rates in the carcass halves.

### Image Processing

All photographic images were assessed for the presence of wild dogs, foxes, feral cats, and any other taxa feeding on carcasses. Individuals could reliably be indentified only for wild dogs (primarily by coat markings) and hence we recorded the number of unique individuals using carcass and transect only for this species. For all wild dogs, foxes and feral cats we recorded the date, time and length (seconds) of visits to carcasses. Each visit was classified into one of four behaviours: (i) feeding, (ii) investigating (animal approached the carcass but did not feed), (iii) scent-marking [40], [65], and (iv) moving through (i.e. none of the other three behaviours). If multiple individuals were recorded at a carcass, then each animal’s activity was recorded separately.

### Statistical Analyses

The behaviour and duration (seconds) of each carcass visit was compiled to describe the daily (i.e. 24 hour, starting and ending at midday) pattern of activity at each carcass. We summarised daily data for each species with 24-hour activity clocks.

We first modelled the factors likely to influence the first visit of a wild dog or fox to each of the 30 carcasses using a discrete-time survival analysis [66], [67]. The models used a truncated Weibull regression with random effects (for transect) in a Bayesian framework. The truncation was due to not all carcasses being visited during the study (i.e. right-censoring). The model was:
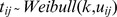


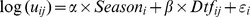


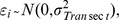
where *t_ij_* is the time that carcass *j* on transect *i* was first visited and *k* and *µ_ij_* are the shape and scale parameters, respectively, for the Weibull distribution. *Season* was winter or spring, *Dtf* the distance to farm (i.e. 0, 1, 2, 3 or 4 km) and 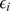 the normally distributed transect-level effects. We fitted this model to our data using a Markov chain Monte Carlo (MCMC) approach implemented in *R* [68] and JAGS [69] using the package *R2jags* [70]. The priors for the model were chosen to represent vague knowledge (Table S1). We used three MCMC chains with over-dispersed starting values and assessed convergence and mixing visually using trace plots and numerically by computing the Gelman-Rubin diagnostic (*R*) [71]. Convergence was defined as *R<*1.05 [71]. After an initial burn-in of 10,000 samples, an additional 10,000 iterations were generated, with every 10^th^ iteration from each chain saved for further inference. The goodness of fit of the model to the data was assessed by comparing the discrepancy of the posterior predictive distribution with the observed data. The posterior predictive distribution consisted of 5000 replicated datasets drawn from the posterior distribution conditioned on the model parameters. The proportion of times the test statistic for the replicated data was greater than or equal to the value for the observed data is the Bayesian *p*-value, with values close to 0 or 1 indicating lack of fit [71].

We next modelled the factors likely to influence the presence/absence of wild dogs and foxes at a carcass within each 24-h period using a discrete-time Markov model in a Bayesian framework [72], [73]. The model was hierarchical, with five carcasses on each transect. We considered five explanatory variables in the model: (i) season (winter or spring), (ii) proportion edible biomass, (iii) distance to farm (km), and (iv) if it had been visited the previous day by the other species (a fox for the dog model and a wild dog for the fox model). We assumed that edible biomass declined linearly between the ∼14-d measurements. The transition matrix was:
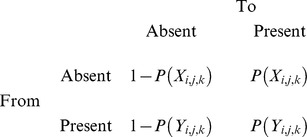


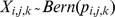


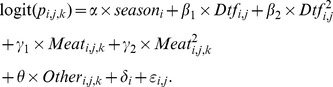



Last, we evaluated potential variables explaining the edible biomass on each carcass at each ∼14-d inspection. Edible biomass values of 100% and 0% were transformed to 0.999 and 0.001, respectively. The logit of edible biomass was fitted using a generalised additive mixed model (GAMM) with a normal distribution [74]. Fixed explanatory variables were: days available (smoothed with a thin plate regression spline) for each season (spring and winter); the total amount of time dogs fed at the carcass to that time; and the total amount of time foxes fed at the carcass to that time. Temporal correlation was included using an autoregressive model with a lag of 1 (i.e. AR1) at the carcass level. Transect was included as a random effect.

### Data and Computer Code

The data and computer code used in our analyses are available from the senior author upon request.

### Ethics Statement

The sambar deer carcasses used in this study were harvested by Melbourne Water employees in accordance with permits issued by Melbourne Water, Melbourne, Australia. Under Australian law, Institutional Animal Ethics Committee approval was not required for this study because all carcasses were from animals harvested for management purposes. Pursuant to the Wildlife Act 1975, this study was conducted under permits 10006257 and 1006612 issued by the Department of Environment and Primary Industries, Melbourne, Australia.

## Results

### Camera Performance

There were no malfunctions of any cameras. However, both cameras were stolen at one carcass during the second interval (i.e. after the first 14 d of photographs had been downloaded) and were not replaced. Our analyses include data from all (i.e. *n* = 30) carcasses unless otherwise stated.

### Deer and Deer Hunters

Sambar deer were either detected by cameras or seen by the study team at all six transects. Fallow deer were detected by cameras at three transects. Deer hunters were detected by cameras at three transects.

### Taxa Detected Feeding on Sambar Deer Carcasses

Nine taxa were detected by our cameras eating sambar deer carcasses: wild dogs, foxes, feral cats, ravens (*Corvus* spp.), wedge-tailed eagles (*Aquila audax*), pied currawongs (*Strepera graculina*), common brush-tailed possums (*Trichosurus vulpecula*), brown goshawks (*Accipiter fasciatus*), and little eagles (*Hieraaetus morphnoides*). However, relative to wild dogs and foxes the seven other taxa consumed negligible biomass and hence were not explicitly included as predictor variables in our analyses of changes in edible carcass biomass (see below).

### Visits and Feeding by Wild Dogs, Foxes and Feral Cats

**Figure 3:**
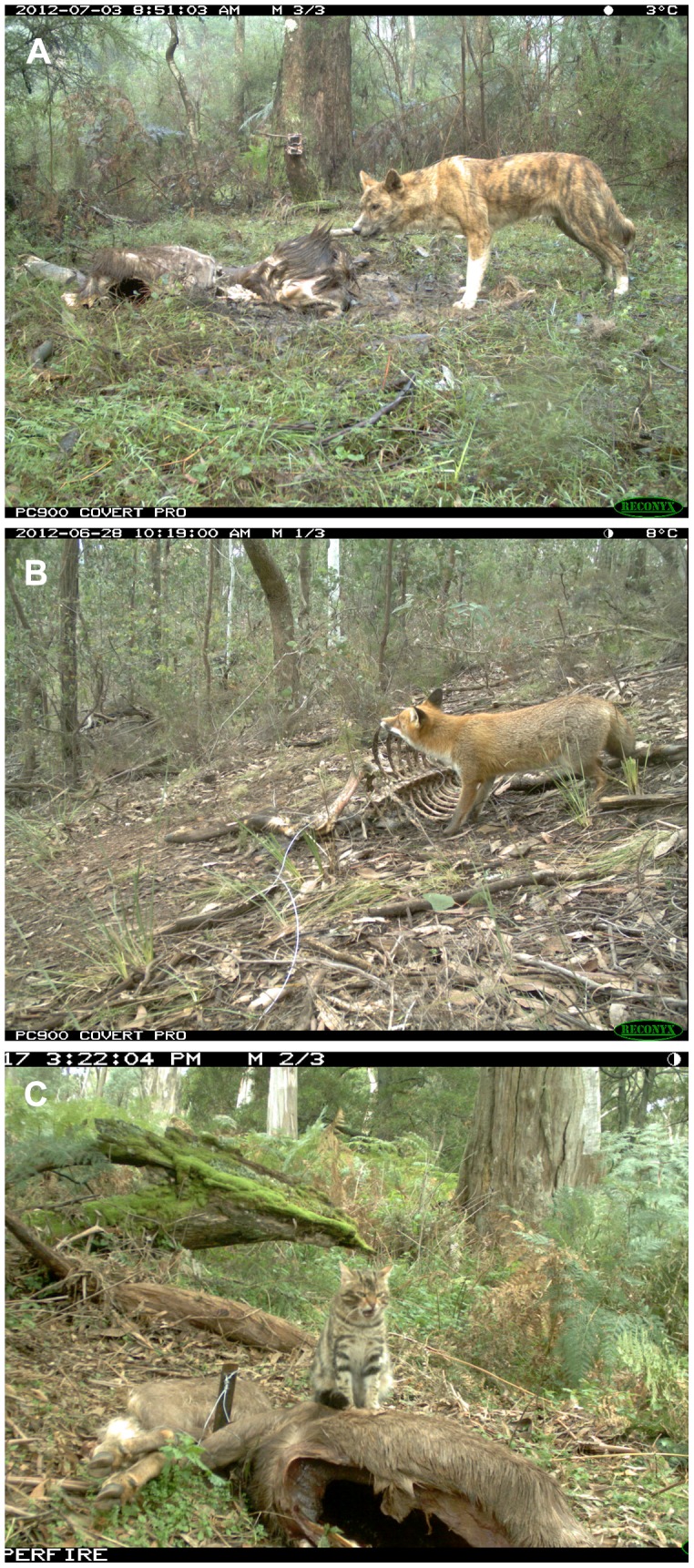
Carnivores feeding on sambar deer carcasses. (a) Wild dog; (b) fox; (c) feral cat. Note the second camera trap on the tree in the background of (a).

**Figure 4:**
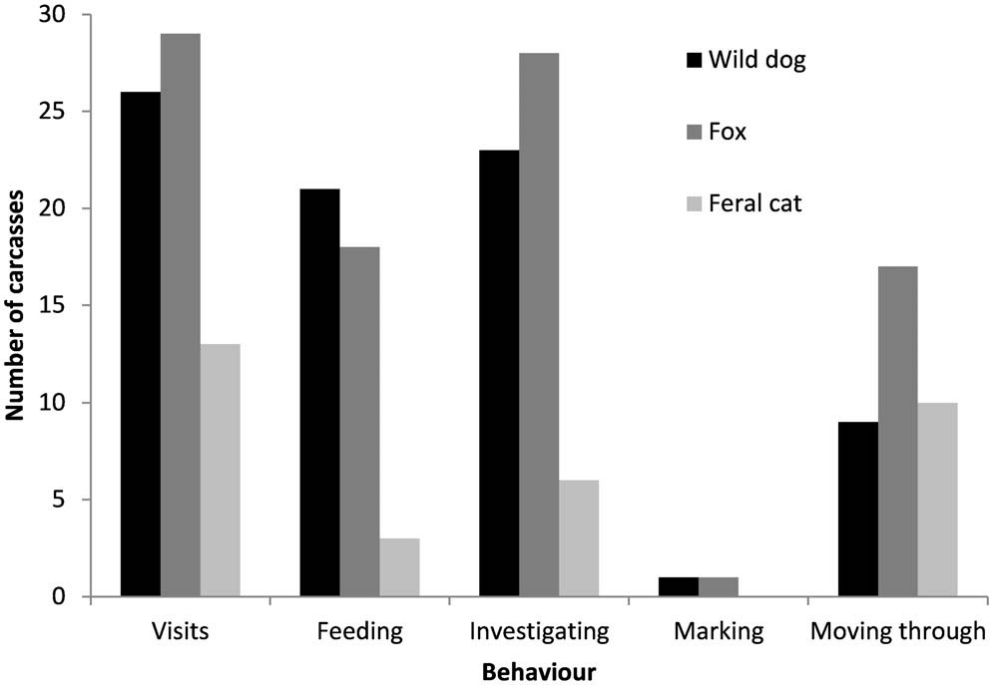
Visits and behaviours of wild dogs, foxes and feral cats at sambar deer carcasses . Behaviours are defined in *Materials and Methods*.

The number of individually recognisable wild dogs detected along the six transects ranged from 3 to 8 (mean ± SD; 5.0±2.0). Wild dogs visited 26 of the 30 carcasses, feeding at 21 carcasses (Figures 3a, 4; Video S1). The modal and mean (±SD) numbers of wild dogs present at a carcass were 1 and 1.2±0.5, respectively.

**Figure 5:**
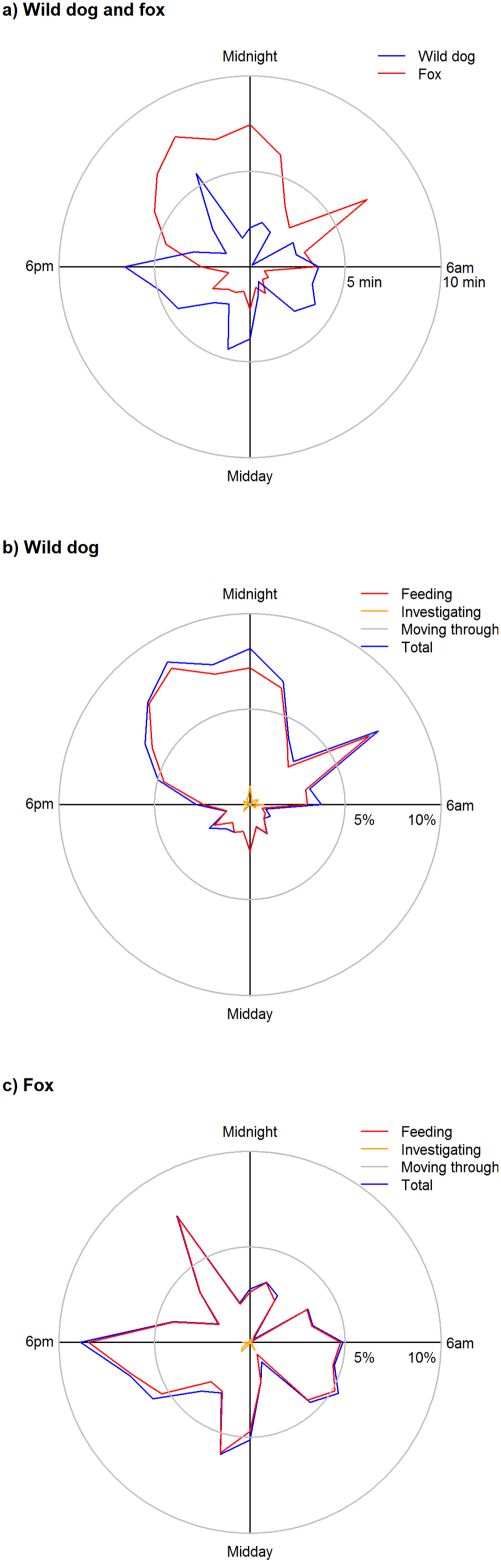
Daily activity patterns of wild dogs and foxes at sambar deer carcasses. (a) Mean total daily activity by wild dogs and foxes, with the inner and outer circles indicating 5 and 10 minutes, respectively. (b) Feeding, investigating and moving through behaviours of wild dogs, with the inner and outer circles indicating 5% and 10% of time, respectively. (c) Feeding, investigating and moving through behaviours of foxes, with the inner and outer circles indicating 5% and 10% of time, respectively.

When >1 wild dog was present at a carcass, on 74% of visits they were all pups and on 9% of visits there was at least one adult and one pup. In contrast to adults, pups sometimes spent long periods of time at carcasses, often involving play behaviour. Wild dogs fed during 57.6% of visits, with feeding bouts lasting for 26.1±33.1 min. The mean (95% CI using adjusted bootstrap percentile method) total amount of time that wild dogs fed at each carcass (*n* = 29; excluding the carcass from which cameras were stolen) was 136.1 min (78.5–242.9 min). Most wild dog activity, including feeding, occurred during 1600–2200 h (Figures 5a, b). However, some feeding also occurred during 0400–0800 h and 1200–1400 h (Figure 5b).

Foxes visited 29 carcasses, feeding at 18 carcasses (Figures 3b, 4; Video S2) and on 48.8% of visits. The modal and mean numbers of foxes present at a carcass were 1 and 1.0±0.05, respectively. When foxes did feed they fed for 22.5±53.6 min. The mean (95% CI using adjusted bootstrap percentile method) total amount of time that foxes fed at each carcass (*n* = 29) was 153.7 min (38.9–594.8 min). Most fox activity, including feeding, occurred during 1900–0100 h (Figures 5a, c). Some feeding also occurred during 0400–0500 h (Figure 5c).

Feral cats visited and fed at 13 and 3 carcasses, respectively (Figures 3c, 4; Video S3). The modal and mean numbers of feral cats present at a carcass were 1 and 1.0±0.00, respectively. The number of carcasses fed on by feral cats was too low to sensibly estimate feeding times and activity, and this species was not considered further in our analyses.

Although multiple wild dogs or foxes were sometimes present at a carcass, the two species were never present together. The closest temporal interaction between these two species at a carcass was one fox leaving at 0202 h, after feeding, followed 10 minutes later by the arrival of wild dog pups. The next closest temporal interaction was a fox feeding at 1008 h prior to an adult wild dog arriving at 1020 h.

### First Visits by Wild Dogs and Foxes

**Figure 6:**
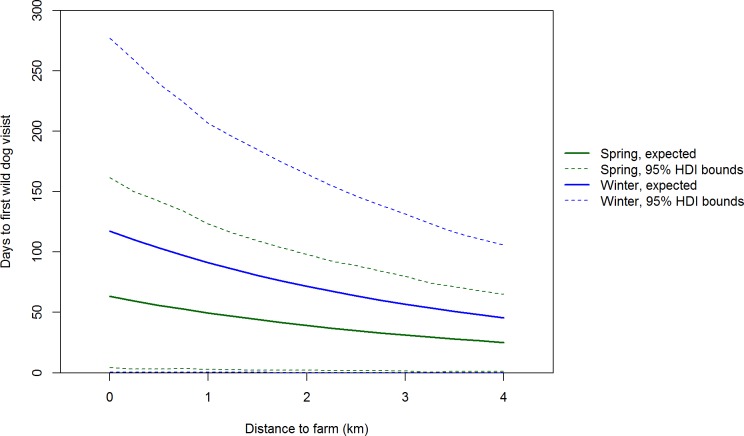
Seasonal relationship between days to first carcass visit by wild dogs and distance to farm. Medians and 95% highest posterior density interval bounds are shown for the winter and spring seasons.

The Bayesian predictive *p*-values for our models of the probability of wild dogs and foxes first visiting a carcass were 0.509 and 0.367, respectively, indicating good fits of the models to the data. The probability of wild dogs and foxes first visiting a carcass increased with the number of days the carcass had been available (i.e. shape parameter 95% credibility intervals are greater than 1) and distance to farm (i.e. 95% credibility intervals for distance to farm are greater than 0) (Tables 1 and 2). The number of days to the first wild dog visit was much lower in spring than winter (ΔDIC = 5.45 for model with season compared to model without season) (Figure 6). The probability of a fox first visiting a carcass increased with the number of days the carcass had been available (Table 2). There was limited support for the probability of a fox first visiting a carcass decreasing with increasing distance from the farm (95% CI for distance to farm included 0), and limited support for the probability of a fox first visiting a carcass being higher in spring compared to winter (ΔDIC = 0.98 for model with season compared to model without season).

**Table 1:** Parameter estimates (and 95% credible intervals) from the model of days to first carcass visit by wild dogs.

Parameter	Median	2.5%	97.5%
Shape (*k*)	1.61	1.12	2.14
Spring constant (*α*_1_)	−5.38	−8.32	−2.81
Winter constant (*α*_1_)	−6.60	−9.77	−3.82
Distance to farm (*β*)	0.38	0.06	0.68
Between-transect variation (*σ* ^2^_Transect_)	1.74		

**Table 2:** Parameter estimates (and 95% credible intervals) from the model of days to first carcass visit by foxes.

Parameter	Median	2.5%	97.5%
Shape (*k*)	1.75	1.21	2.25
Spring constant (*α*_1_)	−6.20	−8.96	−3.18
Winter constant (*α*_1_)	−5.35	−8.75	−2.37
Distance to farm (*β*)	0.08	−0.22	0.38
Between-transect variation (*σ* ^2^_Transect_)	1.93		

### Daily Visits by Wild Dogs and Foxes

**Figure 7:**
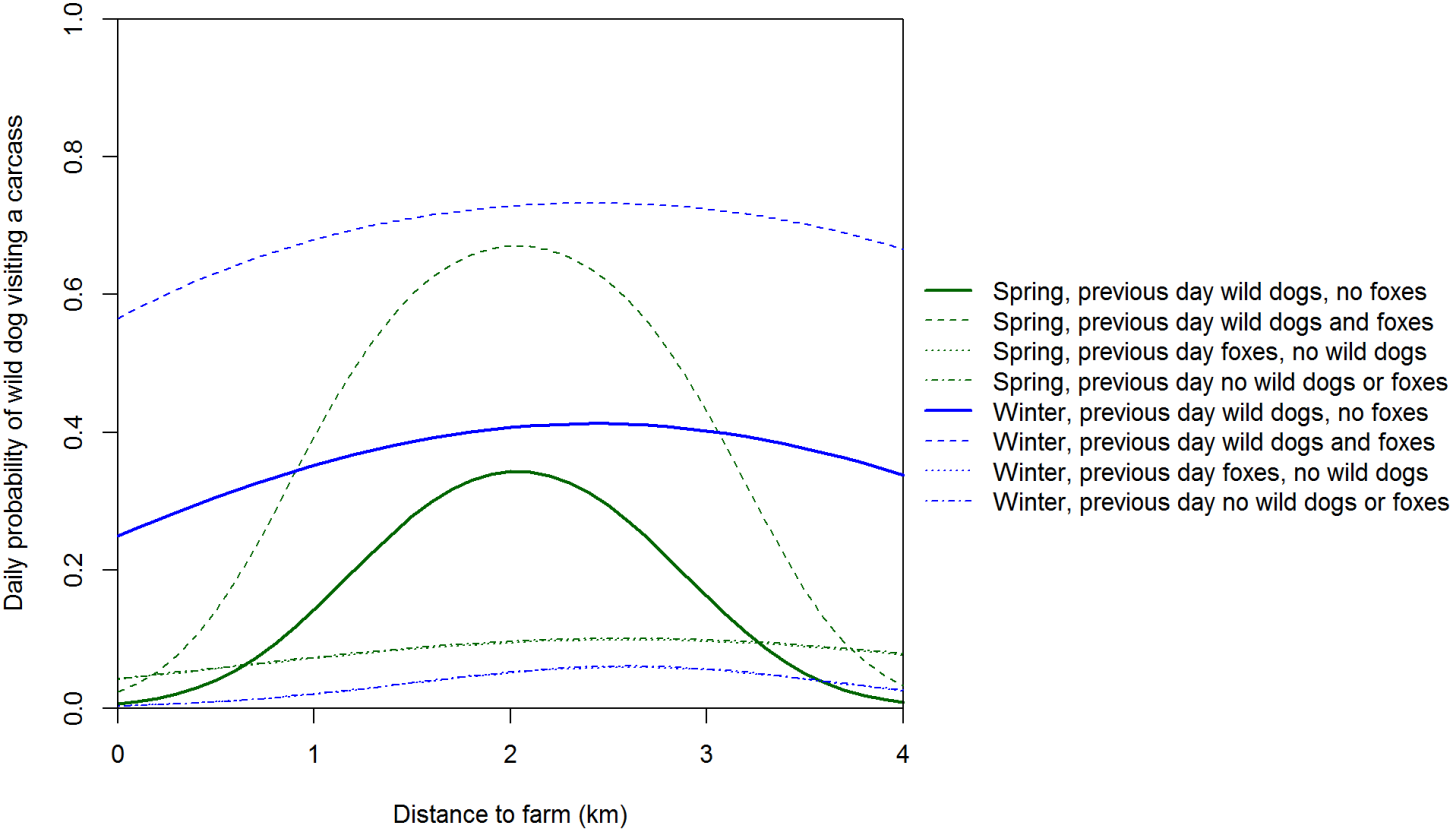
Expected daily probability of a wild dog visiting a sambar deer carcass. Probabilities are medians from the posterior distribution with average edible biomass.

**Figure 8:**
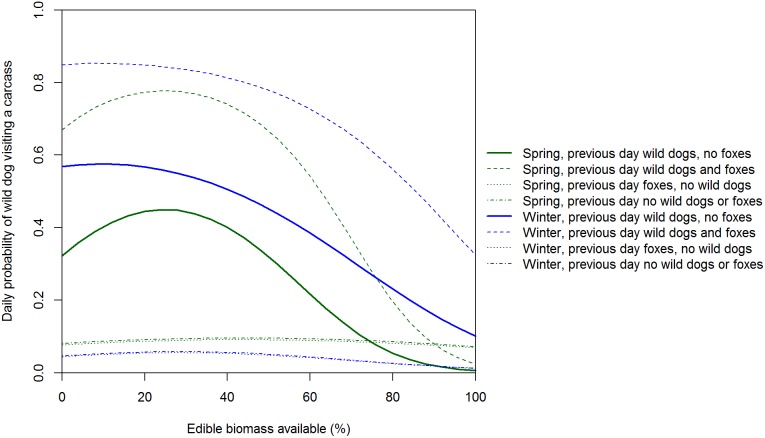
Expected daily probability of a wild dog visiting a carcass as a function of edible biomass. Expected probabilities are medians from the posterior distribution at a carcass 2/or fox.

The residuals of our discrete-time Markov model of factors influencing the daily presence/absence of wild dogs at carcasses showed no systematic variation, but the Bayesian *p*-value of 0.066 indicates that this model was not a good fit to the data. However, attempts to improve the model fit were unsuccessful and since the model for foxes was a reasonable fit to the data (see below) we used the wild dog model for inference. Wild dogs were more likely to visit a carcass if they had visited the previous day than if they had not visited the previous day (Table 3). The probability of a wild dog visiting a carcass had a convex relationship with distance to farm in all seasons, but particularly in winter if dogs were not present the previous day and in spring if dogs were present the previous day (Figure 7). Wild dog visits were lowest at carcasses 1 and 5 (i.e. 0.05 and 4 km from farms) and highest at carcasses 3 and 4 (i.e. 2 and 3 km from farms). The amount of edible biomass was important to all previous states and seasons, with the probability of a visit generally decreasing strongly as edible biomass declined (Figure 8). Interestingly, the presence of wild dogs and foxes at a carcass the previous day increased the odds of wild dog presence by a factor of 4.

**Table 3:** Parameter estimates for the discrete-time Markov models of wild dog presence at a sambar deer carcass during a 24-h period.

Previous State	Season	Variable	Estimate		
			Median	2.75%	97.5%
Dogs not present	Either	Fox present previous day	−0.025	−0.779	0.661
	Spring	Intercept	−2.231	−4.399	0.112
		Distance to farm	0.162	−0.092	0.429
		Distance to farm^2^	−0.137	−0.369	0.092
		Edible biomass available	−0.032	−0.346	0.306
		Edible biomass available^2^	−1.077	−1.555	−0.569
	Winter	Intercept	−0.650	−3.336	1.869
		Distance to farm	0.076	−0.774	0.911
		Distance to farm^2^	−1.067	−2.025	−0.231
		Edible biomass available	−1.574	−3.524	0.240
		Edible biomass available^2^	−2.220	−4.427	−0.093
Dogs present	Either	Fox present previous day	1.361	0.189	2.649
	Spring	Intercept	−2.894	−5.000	−0.475
		Distance to farm	0.527	0.133	0.930
		Distance to farm^2^	−0.445	−0.737	−0.132
		Edible biomass available	−0.588	−0.971	−0.195
		Edible biomass available^2^	−0.268	−0.766	0.236
	Winter	Intercept	−0.375	−2.709	2.148
		Distance to farm	0.106	−0.576	0.868
		Distance to farm^2^	−0.127	−0.671	0.454
		Edible biomass available	−1.121	−2.259	−0.117
		Edible biomass available^2^	−1.681	−3.023	−0.456
Transect variance			2.547	0.120	11.763
Carcass variance			0.137	0.000	0.584

The superscripts in ‘Distance to farm^2^’ and ‘Edible biomass available^2^’ are quadratic terms.

**Figure 9:**
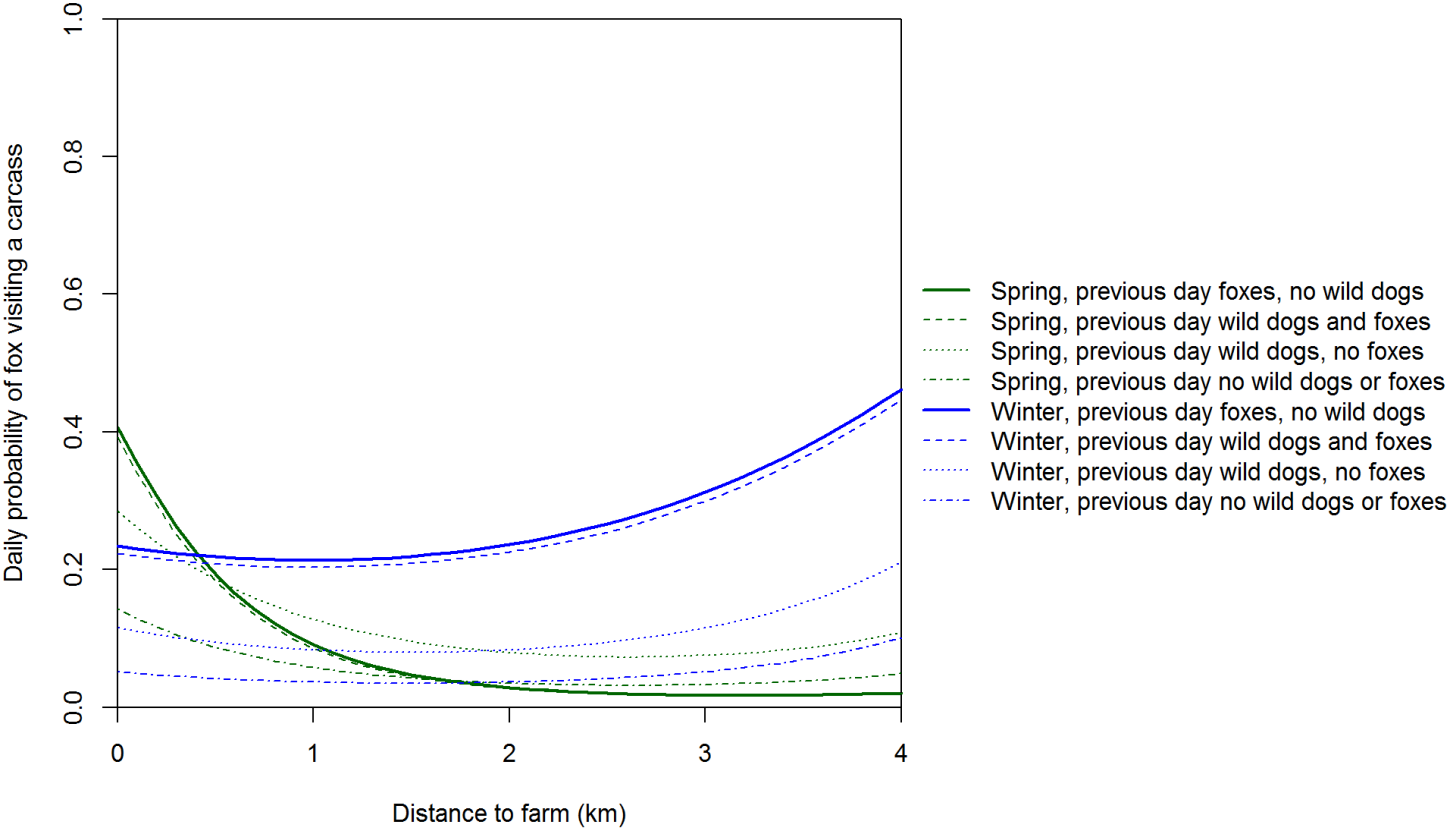
Expected daily probability of a fox visiting a carcass as a function of distance to farm. Expected probabilities are medians from the posterior distribution with average edible biomass and are shown for all combinations of season and the previous presence of wild dog and/or fox.

**Figure 10:**
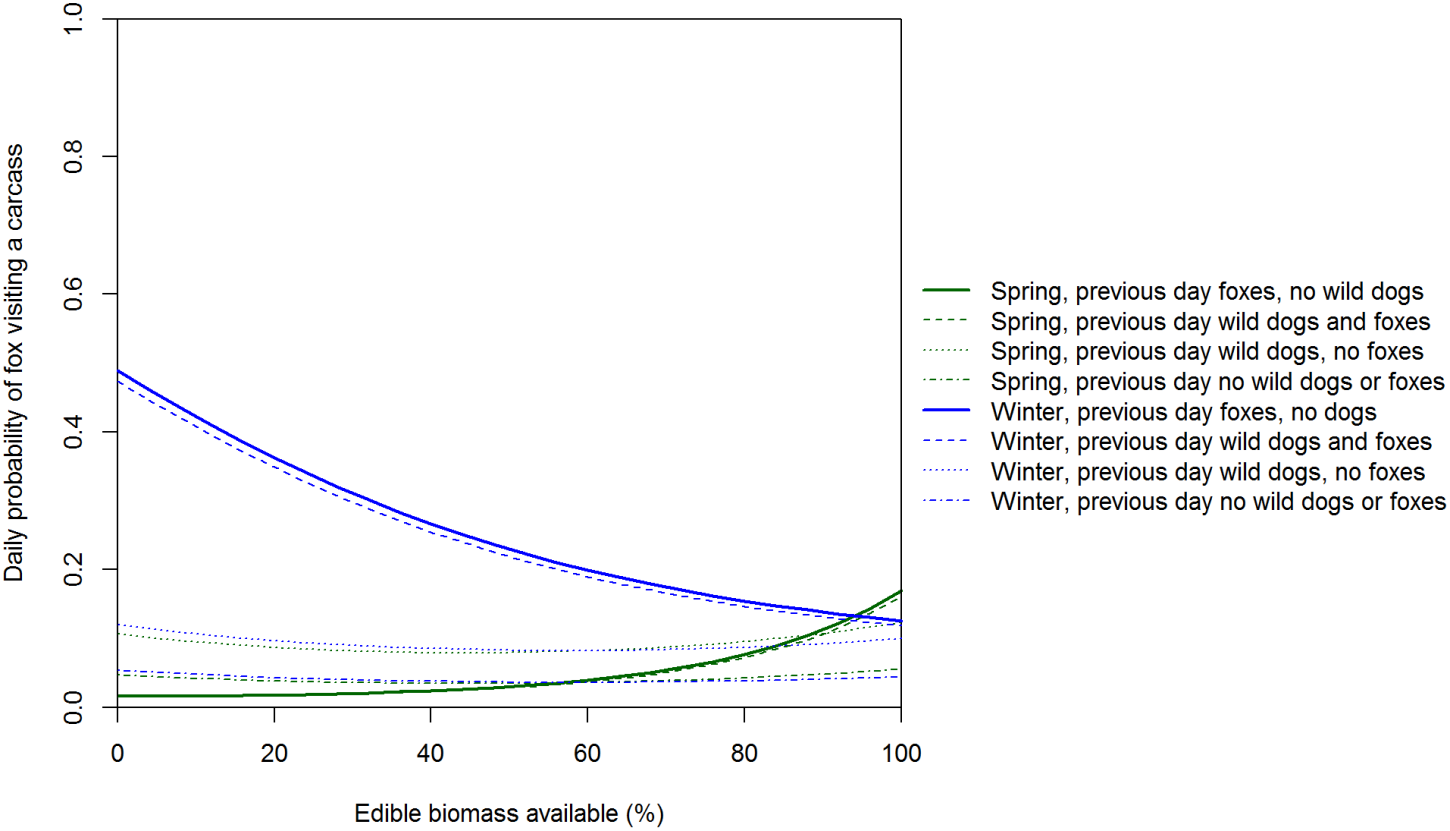
Expected daily probability of a fox visiting a carcass as a function of edible biomass. Expected probabilities are medians from the posterior distribution at a carcass 2/or fox.

The residuals of our model of factors influencing the daily presence/absence of foxes at carcasses showed no systematic variation and the Bayesian *p*-value of 0.198 indicated a reasonable fit to the data. The spatial pattern of fox visits to carcasses varied with season but was usually concave, with most fox visits at carcasses 1 and 5 (i.e. nearest and 4 km from farms) and fewest visits at carcass 3 (i.e. 2 km from farms; Table 4; Figure 9). Visits to carcasses by foxes varied between seasons and were ‘significantly’ higher in winter if foxes had been present the day prior than if foxes had not been present (Table 4; Figures 9 and 10). Given that a fox was present the previous day, the odds of a fox visit increased in winter and decreased in spring as edible biomass declined (Figure 9). The daily odds of foxes being present at a carcass increased by a factor of almost 2.4 given dogs were present the previous day but foxes were not (Table 4).

**Table 4:** Parameter estimates for the discrete-time Markov models of fox presence at a sambar deer carcass during a 24-h period.

Previous State	Season	Variable	Estimate		
			Median	2.75%	97.5%
Foxes not present	Either	Dog present previous day	0.869	0.270	1.501
	Spring	Intercept	−3.321	−4.615	−1.929
		Distance to farm	−0.295	−0.653	0.097
		Distance to farm^2^	0.235	−0.093	0.561
		Edible biomass available	0.041	−0.218	0.299
		Edible biomass available^2^	−0.363	−0.780	0.008
	Winter	Intercept	−3.271	−4.625	−2.069
		Distance to farm	0.180	−0.209	0.561
		Distance to farm^2^	0.181	−0.151	0.514
		Edible biomass available	−0.093	−0.365	0.222
		Edible biomass available^2^	−0.015	−0.404	0.361
Foxes present	Either	Dog present previous day	−0.061	−1.014	0.952
	Spring	Intercept	−3.526	−5.359	−1.736
		Distance to farm	−0.869	−1.596	−0.239
		Distance to farm^2^	0.352	−0.121	0.838
		Edible biomass available	0.937	0.444	1.447
		Edible biomass available^2^	0.449	−0.298	1.135
	Winter	Intercept	−1.173	−2.650	0.316
		Distance to farm	0.257	−0.177	0.725
		Distance to farm^2^	0.126	−0.279	0.537
		Edible biomass available	−0.735	−1.237	−0.283
		Edible biomass available^2^	−0.506	−1.206	0.144
Transect variance			0.372	0.000	2.569
Carcass variance			0.856	0.338	1.843

The superscripts in ‘Distance to farm^2^’ and ‘Edible biomass available^2^’ are quadratic terms.

### Impact of Wild Dogs and Foxes on Edible Carcass Biomass

**Figure 11:**
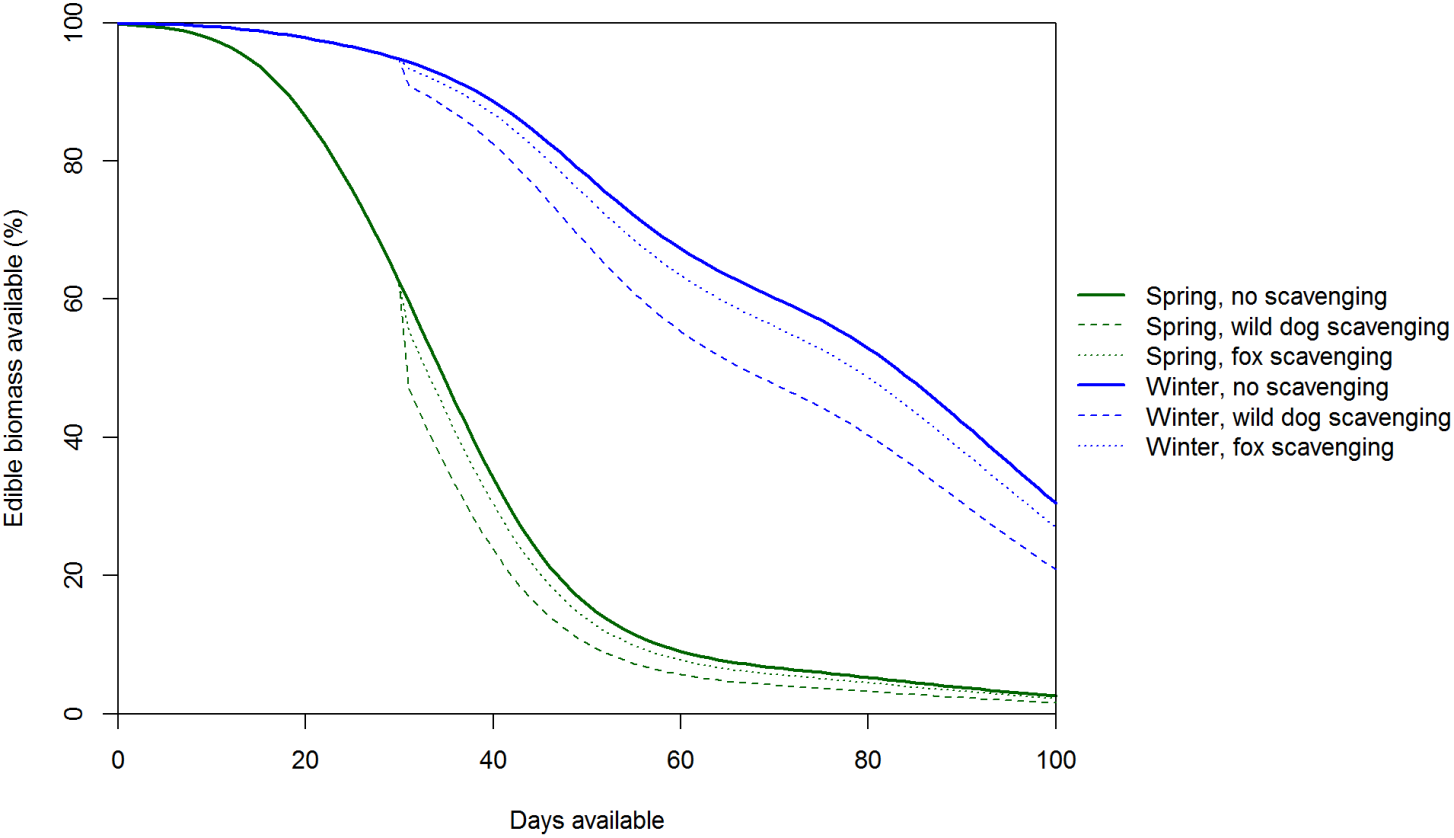
Temporal changes in the edible biomass of carcasses as a function of season and carnivory. The effects of feeding by wild dogs and foxes are illustrated by including 180(136 minutes) and foxes (154 minutes) spent feeding at a carcass and enabled the effects of foxes to be visible.

The Pearson residuals for the GAMM estimating the impact of wild dogs and foxes on the amount of edible biomass on carcasses showed no pattern and were all within 4 units. Hence, there was no apparent violation of model assumptions. The model explained a substantial amount of variation (*R* ^2^ = 0.736). There was strong evidence that edible biomass declined at a slower rate in winter than spring (*P*<0.001 for the smoothed time term and *P* = 0.002 for the additional time term for winter), with ≤10% of edible biomass remaining after 11 weeks for carcasses placed out in spring compared to >14 weeks for carcasses placed out in early winter (Figure 11). The amount of time that wild dogs (*P*<0.001) and foxes (*P* = 0.020) spent feeding had significant negative effects on edible biomass (Table 5), although the effects of feeding by either species were small relative to season (Figure 11).

**Table 5:** Parameter estimates for the model of temporal change in the amount of edible biomass on sambar deer carcasses during winter and spring.

Parameter	Estimate	Standard error	*P*-value
Intercept	−0.521	0.569	0.361
Time wild dogs feeding	−0.530	0.147	<0.001
Time foxes feeding	−0.326	0.139	0.020
Within-transect variance	0.842		

## Discussion

The carcasses of large ungulates such as sambar deer represent a potentially substantial food source for mammalian carnivores [20], [21], [75]. Hunters in Victoria harvest c. 32,000 sambar deer annually, and since there is no requirement for hunters to remove or bury the carcass many will be available to scavengers. Wild dogs and foxes fed on most sambar deer carcasses, but they seldom remained at carcasses for long and had a smaller effect on the loss of edible biomass than we expected. Feral cats seldom fed on sambar deer carcasses, a result consistent with the belief that this species is an obligate predator in Australia (i.e. prefers live prey [37]).

Wild dog visits peaked at carcasses 2 and 3 km from farms (i.e. a convex relationship with distance to farm). The low use of carcasses 0.05 and 1 km from farms is likely a legacy of intensive control of this species on farms and up to 3 km into adjacent public land within Victoria. In a similar New South Wales landscape, satellite tracking revealed that wild dogs seldom used private land with domestic livestock and the one wild dog that did was shot [76]. We are uncertain why there were so few wild dog visits to carcasses 4 km from farms. One hypothesis is that hunters may seldom venture this far into forest and hence wild dogs were unaccustomed to encountering hunter-shot deer carcasses in this part of the landscape and therefore avoided them. Further study of how wild dogs utilise forested landscapes in south-eastern Australia may better explain the spatial pattern of carcass visits and feeding. Wild dogs can kill foxes [42] and there is evidence of foxes avoiding sites used by wild dogs [41], [44]. If there is interference competition and/or intraguild predation, then we would expect negative spatial and temporal relationships between wild dogs and foxes [43]. In contrast to wild dogs, fox visits peaked at carcasses nearest and furthest from farms (i.e. a concave relationship with distance to farm). The spatial pattern of carcass use by foxes was therefore consistent with the hypothesis that foxes actively avoid wild dogs [41]–[43]: fox visits peaked at carcasses where wild dog visits were least frequent. We suggest that in the absence of intensive, sustained long-term control, wild dog densities (and hence visits to sambar deer carcasses) would likely be much higher at and near the farm-forest interface because of the high food availability there [7], but that fox visits would be lower at and near the farm-forest interface.

Daily activity patterns were also consistent with the hypothesis that foxes actively avoid wild dogs. Wild dogs visited carcasses throughout the 24-hour period, but activity peaked at dusk and dawn, consistent with previous studies [63], [77]. In contrast, foxes are mostly nocturnal [78] and visited carcasses almost exclusively from 1900−0430 (i.e. after dusk and before dawn). Consistent with the idea that foxes actively avoid wild dogs, the two species were never observed together at a carcass. Our Markov models of the factors influencing the daily visits of wild dogs and foxes showed interesting temporal interactions between these species. The presence of wild dogs and foxes the previous day increased the daily odds of wild dogs visiting a carcass. Wild dogs may have been attracted to carcasses by olfactory cues left by foxes. Although marking behaviour by foxes was rarely detected by our cameras, these olfactory cues may have been left outside the field of view of our cameras. Wild dogs may ‘defend’ the carcass from foxes, an activity that could result in foxes being killed by wild dogs. Our finding that the presence of wild dogs the previous day increased the daily odds of foxes visiting a carcass raises the intriguing possibility that foxes may have been attracted to carcasses where wild dogs had fed the previous night by the increased availability of ‘scraps’ left by wild dogs feeding. In Manitoba, coyotes (*Canis latrans*) frequently followed wolves and scavenged at their kills [23]. Given that wild dogs can kill foxes, following wild dogs with a delay of 24 h would be a less risky strategy for foxes than shadowing wild dogs at food resources. There is clearly much to learn about the fine-scale spatial and temporal partitioning of resources by wild dogs and foxes in Australia.

Mammalian scavengers compete with microbes and insects for the edible biomass of carcasses [60]. Sambar deer carcasses lost edible biomass faster in spring than winter, likely due to increased maggot and microbial activity in the warmer months. Sambar deer carcasses placed out in winter, particularly at higher elevations, lost little or no edible biomass to maggot and microbial activity until the warmer spring months. Carcasses subject to intensive feeding by wild dogs and/or foxes were eventually dismembered, with bones moved up to 20 m. In contrast, carcasses subject to intensive maggot and microbial activity quickly putrefied and dried such that they were encased in a hard envelope of desiccated skin (‘mummification’ [79]). Mummified carcasses can persist for many years in dry environments [75]. The date of harvesting will therefore strongly influence the length of time that ungulate carcasses are available to mammalian scavengers (rather than microbial and insect decomposers) in south-eastern Australia.

Camera traps are revolutionising the study of animal ecology and behaviour [80]. The date- and time-stamping of photographs enables temporal activity to be evaluated at a variety of scales [61]. Most Northern Hemisphere studies have made inferences about the use of carcasses by mammalian carnivores using tracks in snow [21], [23], [28], [64]. Most Australian studies have used sand plots rather than camera traps to investigate interactions among carnivores (e.g. refs [41], [43], [45], [81] but see refs [44], [46], [82]). The key advantage of cameras such as those used in our study is that they continuously monitor (barring theft or malfunction) different activities, including feeding (Videos S1–S3). Our cameras revealed that only half of wild dog and fox visits to carcasses involved feeding, suggesting that estimates of carcass use based on signs such as tracks may be significantly positively biased. We encourage other researchers to use camera traps to investigate spatio-temporal interactions within carnivore guilds at focal sites (e.g. food, water and movement corridors), including within Australia where there is debate about the extent of mesopredator release among wild dogs, foxes and feral cats [83], [84] and interest in the extent that anthropogenic resources such as artificial water points, boneyards and refuse pits subsidise mammalian carnivores [12], [81].

Although wild dog pups sometimes spent long periods at sambar deer carcasses, we were surprised that wild dogs did not spend more time feeding at sambar deer carcasses and did not contribute more to the removal of edible biomass from carcasses. There are several possible explanations for this. First, our study sites had been subject to wild dog control for many years and hence wild dog abundances were likely low, particularly close to farms (see above), relative to what they would be in the absence of control. Low frequencies of visits to carcasses by wild dogs, particularly adults, suggest that this species was at low density. Wild dogs form large packs in the absence of control and the presence of abundant food, with small packs considered a product of control [7]. Second, the availability of more preferred alternative prey may have meant that wild dogs did not ‘need’ to eat sambar deer carcasses. The diet of wild dogs in south-eastern Australia is dominated by macropods and wombats [85]–[87]. The low rate of visits involving feeding supports this hypothesis. Indeed, Fleming et al. [7] considered wild dogs to be “specialist” hunters rather than “opportunistic generalists”. Third, and related to the previous point, the spatially and temporally unpredictable distribution of carcasses in the landscape means that wild dogs (and foxes) may have been using these areas less than other parts of the landscape. However, our carcasses were always within 50 m of roads and tracks, which are thought to be important movement corridors for wild dogs and foxes in south-eastern Australian forests [88]. Fourth, hunter-shot deer carcasses unrelated to our study (and therefore unknown to us) may have been present in the study area. Hunting was permitted throughout our study area for the duration of our study and hence wild dogs and foxes may have been utilising other hunter-shot deer carcasses. Fifth, sambar deer carcasses rapidly decomposed during the warmer spring season such that virtually all edible biomass had been removed after 11 weeks. Hence, the flesh of carcasses remained available to all carnivores for longer during winter than spring. If we had monitored carcasses in only one season rather than two seasons then our estimates of the utilisation of carcasses by wild dogs, foxes and feral cats would have been different.

## Conclusion

Spatial and temporal patterns of visits to sambar deer carcasses by mammalian carnivores in south-eastern Australia were consistent with the hypothesis that foxes avoid wild dogs. Wild dog activity peaked at carcasses 2 and 3 km from farms, a likely legacy of wild dog control, whereas fox activity peaked at carcasses nearest and 4 km from farms. Wild dog activity peaked at dawn and dusk, whereas nearly all fox activity occurred after dusk and before dawn. Feral cats seldom fed on sambar deer carcasses, consistent with the belief that this species is an obligate predator in Australia.

Although most sambar deer carcasses were fed on by wild dogs and foxes, their feeding activities did not greatly accelerate the decomposition of carcasses. Instead, most carcasses decomposed through insect and microbial activity in warmer weather. The extent to which hunter-shot ungulate carcasses will be consumed by mammalian carnivores will depend upon the abundance of carnivores, spatial location, season and the availability of more preferred food resources.

## Supporting Information

Table S1 **Prior distributions for the parameters of the model of time to first carcass visit.**
(DOC)

Video S1 **Video (30 sec) of a wild dog feeding on a sambar deer carcass recorded by a Scoutguard SG550v Infrared Digital Scouting Camera (HCO, Norcross, Georgia, USA).**
(WMV)

Video S2 **Video (30 sec) of a fox feeding on a sambar deer carcass recorded by a Scoutguard SG550v Infrared Digital Scouting Camera (HCO, Norcross, Georgia, USA).**
(WMV)

Video S3 **Video (30 sec) of a feral cat feeding on a sambar deer carcass recorded by a Scoutguard SG550v Infrared Digital Scouting Camera (HCO, Norcross, Georgia, USA).**
(WMV)

## References

[1] Crooks KR, Soulé ME (1999) Mesopredator release and avifaunal extinctions in a fragmented system. Nature 400: 563–566.

[2] Beschta RL, Ripple WJ (2009) Large predators and trophic cascades in terrestrial ecosystems of the western United States. Biol Conserv 142: 2401–2414.

[3] Ritchie EG, Johnson CN (2009) Predator interactions, mesopredator release and biodiversity conservation. Ecol Lett 12: 982–998. 19614756 10.1111/j.1461-0248.2009.01347.x

[4] Ripple WJ, Estes JA, Beschta RL, Wilmers CC, Ritchie EG, et al (2014) Status and ecological effects of the world’s largest carnivores. Science 343: 1241484. 24408439 10.1126/science.1241484

[5] Frump RR (2006) The man-eaters of Eden: life and death in Kruger National Park. Guilford: Lyons Press. 216 p.

[6] Muhly TB, Musiani M (2009) Livestock depredation by wolves and the ranching economy in the Northwestern U.S. Ecol Econ. 68: 2439–2450.

[7] Fleming P, Corbert L, Harden R, Thomson P (2001) Managing the impact of dingoes and other wild dogs. Canberra: Bureau of Rural Science. 186 p.

[8] Pierce BM, Bleich VC, Bowyer RT (2000) Social organization of mountain lions: does a land-tenure system regulate population size? Ecology 81: 1533–1543.

[9] Morehouse AT, Boyce MS (2011) From venison to beef: seasonal changes in wolf diet composition in a livestock grazing landscape. Front Ecol Environ 9: 440–445.

[10] Yirga G, De longh HH, Leirs H, Gebrihiwot K, Deckers J, et al (2012) Adaptability of large carnivores to changing anthropogenic food sources: diet change of spotted hyena (*Crocuta crocuta*) during Christian fasting period in northern Ethiopia. J Anim Ecol 81: 1052–1055. 22486435 10.1111/j.1365-2656.2012.01977.x

[11] Newsome TM, Ballard G-A, Dickman CR, Fleming PJS, van de Ven R (2013) Home range, activity and sociality of a top predator, the dingo: a test of the Resource Dispersion Hypothesis. Ecography 36: 914–925.

[12] Newsome TM, Ballard G-A, Dickman CR, Fleming PJS, Howden C (2013) Anthropogenic resource subsidies determine space use by Australian Arid Zone dingoes: an improved resource selection modeling approach. PLOS One 8(5): e63931. 23750191 10.1371/journal.pone.0063931 PMC3667862

[13] Saunders G, Coman BJ, Kinnear J, Braysher M (1995) Managing vertebrate pests: Foxes. Canberra: Australian Government Publishing Service. 141.

[14] Cortés-Avizanda A, Selva N, Carrete M, Donázar JA (2009) Effects of carrion resources on herbivore spatial distribution are mediated by facultative scavengers. Basic Appl Ecol 10: 265–272.

[15] Peterson RO, Ciucci P (2003) The wolf as a carnivore. In: Mech LD, Boitani L, editors. Wolves: behaviour, ecology and conservation. Chicago: University of Chicago Press. 104–130.

[16] Mattioli S (2011) Family Cervidae (Deer). In: Wilson DE, Mittermeier RA, editors. Handbook of the mammals of the world. 2. Hoofed mammals. Barcelona: Lynx Edicions. 350–443.

[17] Apollonio M, Andersen R, Putman R (2010) European ungulates and their management in the 21^st^ Century. Cambridge: Cambridge University Press. 604 p.

[18] Moriarty A (2004) The liberation, distribution, abundance and management of wild deer in Australia. Wildl Res 31: 291–299.

[19] Gordon IJ, Hester AJ, Festa-Bianchet M (2004) The management of wild large herbivores to meet economic, conservation and environmental objectives. J Appl Ecol 41: 1021–1031.

[20] Wilmers CC, Stahler DR, Crabtree RL, Smith DW, Getz WM (2003) Resource dispersion and consumer dominance: scavenging at wolf- and hunter-killed carcasses in Greater Yellowstone, USA. Ecol Lett 6: 996–1003.

[21] Selva N, Jedrzejewska B, Jedrzejewski W, Wajrak A (2005) Factors affecting carcass use by a guild of scavengers in European temperate woodland. Can J Zool 83: 1590–1601.

[22] Dickman CR (1992) Commensal and mutualistic interactions among terrestrialvertebrates. Trends Ecol Evol 7: 194–197. 21236006 10.1016/0169-5347(92)90072-J

[23] Paquet PC (1992) Prey use strategies of sympatric wolves and coyotes in Riding Mountain National Park, Manitoba. J Mammal 73: 337–343.

[24] Hunter JS, Durant SM, Caro TM (2007) To flee or not to flee: predator avoidance by cheetahs at kills. Behav Ecol Sociobiol 61: 1033–1042.

[25] Macdonald DW (1983) The ecology of carnivore social behavior. Nature 301: 379–384.

[26] Creel S, Spong G, Creel N (2001) Interspecific competition and the population biology of extinction-prone carnivores. In: Gittleman JL, Funk SM, Macdonald DW, Wayne RK, editors. Carnivore conservation. Cambridge: Cambridge University Press. 35–60.

[27] Fedriani JM, Fuller TK, Sauvajot RM, York EC (2000) Competition and intraguild predation among three sympatric carnivores. Oecologia 125: 258–270. 24595837 10.1007/s004420000448

[28] Selva N, Fortuna MA (2007) The nested structure of a scavenger community. Proc Roy Soc B 274: 1101–1108. 10.1098/rspb.2006.0232 PMC2124470 17301021

[29] Read JL, Wilson D (2004) Scavengers and detritovores of kangaroo harvest offcuts in arid Australia. Wildl Res 31: 51–256.

[30] Wallach AD, Ritchie EG, Read J, O’Neill AJ (2009) More than mere numbers: the impact of lethal control on social stability in a top-order predator. PLoS One 4: e6861. 19724642 10.1371/journal.pone.0006861 PMC2730570

[31] Savolainen P, Leitner T, Wilton AN, Matisoo-Smith E, Lundeberg J (2004) A detailed picture of the origin of the Australian dingo, obtained from the study of mitochondrial DNA. Proc Natl Acad Sci U S A 101: 12387–12390. 15299143 10.1073/pnas.0401814101 PMC514485

[32] Pople AR, Grigg GC, Cairns SC, Beard LA, Alexander P (2000) Trends in numbers of kangaroos and emus on either side of the South Australian dingo fence: evidence for predator regulation? Wildl Res 27: 269–276.

[33] Letnic M, Koch F, Gordon C, Crowther MS, Dickman CR (2009) Keystone effects of an alien top-predator stem extinctions of native mammals. Proc Roy Soc B 276: 3249–3256. 10.1098/rspb.2009.0574 PMC2817164 19535372

[34] Letnic M, Crowther MS (2012) Patterns in the abundance of kangaroo populations in arid Australia are consistent with the exploitation ecosystems hypothesis. Oikos 121: 761–769.

[35] Colman NJ, Gordon CE, Crowther MS, Letnic M (2014) Lethal control of an apex predator has unintended cascading effects on forest mammal assemblages. Proc Roy Soc B 281: 20133094. 10.1098/rspb.2013.3094 PMC3973261 24619441

[36] Victoria Government (2013) Victoria Government Gazette No. G 39 Thursday 26 September 2013. Melbourne: Victorian Government Printer. 36 p.

[37] Dickman CR (1996) Overview of the impacts of feral cats on Australian native fauna. Canberra: Australian Nature Conservation Agency. 92 p.

[38] Johnson CN, Isaac JL, Fisher DO (2007) Rarity of a top predator triggers continent-wide collapse of mammal prey: dingoes and marsupials in Australia. Proc Roy Soc B 274: 341–346. 10.1098/rspb.2006.3711 PMC1702374 17164197

[39] Saunders GR, Gentle MN, Dickman CR (2010) The impacts and management of foxes *Vulpes vulpes* in Australia. Mamm Rev 40: 181–211.

[40] Corbett LK (1995) The dingo in Australia and Asia. Sydney: New South Wales University Press. 200 p.

[41] Mitchell BD, Banks PB (2005) Do wild dogs exclude foxes? Evidence for competition from dietary and spatial overlaps. Austral Ecol 30: 581–591.

[42] Moseby KE, Neilly H, Read JL, Crisp HA (2012) Interactions between a top order predator and exotic mesopredators in the Australian Rangelands. Int J Ecol 250352 (doi:10.1155/2012/250352).

[43] Johnson CN, VanDerWal J (2009) Evidence that dingoes limit abundance of a mesopredator in eastern Australian forests. J Appl Ecol 46: 641–646.

[44] Brook LA, Johnson CN, Ritchie EG (2012) Effects of predator control on behaviour of an apex predator and indirect consequences for mesopredator suppression. J Appl Ecol 49: 1278–1286.

[45] Kennedy M, Phillips BL, Legge S, Murphy SA, Faulkner RA (2012) Do dingoes suppress the activity of feral cats in northern Australia? Austral Ecol 37: 134–139.

[46] Wang Y, Fisher DO (2012) Dingoes affect activity of feral cats, but do not exclude them from the habitat of an endangered macropod. Wildl Res 39: 611–620.

[47] Leslie DM Jr (2011) *Rusa unicolor* (Artiodactyla: Cervidae). Mammalian Species 43: 1–30.

[48] Bentley A (1998) An introduction to the deer of Australia. Melbourne: Australian Deer Research Foundation. 367 p.

[49] Van Dyck S, Strahan R (2008) Mammals of Australia. Third edition. Melbourne CSIRO Publishing. 888 p.

[50] Downes M (1983) The forest deer project 1982. Vol. II. Ecology and hunting. Melbourne: Forests Commission. 65 p.

[51] Menkhorst PW (1995) Mammals of Victoria: Distribution, ecology and conservation. Melbourne: Oxford University Press. 359 p.

[52] Gormley AM, Forsyth DM, Griffioen P, Woodford L, Lindeman M, et al (2011) Using presence-only and presence-absence data to estimate the current and potential distributions of established invasive species. J Appl Ecol 48: 25–34. 21339812 10.1111/j.1365-2664.2010.01911.x PMC3038347

[53] Forsyth DM, McLeod SR, Scroggie MP, White M (2009) Modelling the abundance of wildlife using field surveys and GIS: non-native sambar deer (*Cervus unicolor*) in the Yarra Ranges, south-eastern Australia. Wildl Res 36: 231–241.

[54] Gormley AM, Turnbull JD (2009) Estimates of harvest for deer, duck and quail in Victoria: results from surveys of Victorian game licence holders in 2009. Arthur Rylah Institute for Environmental Research Technical Report Series No. 196. Heidelberg: Department of Sustainability and Environment. 24 p.

[55] Gormley AM, Turnbull JD (2010) Estimates of harvest for deer, duck and quail in Victoria: results from surveys of Victorian game licence holders in 2010. Arthur Rylah Institute for Environmental Research Technical Report Series No. 210. Heidelberg: Department of Sustainability and Environment. 23 p.

[56] Gormley AM, Turnbull JD (2011) Estimates of harvest for deer, duck and quail in Victoria: results from surveys of Victorian game licence holders in 2011. Arthur Rylah Institute for Environmental Research Technical Report Series No. 224. Heidelberg: Department of Sustainability and Environment. 26 p.

[57] Moloney PD, Turnbull JD (2012) Estimates of harvest for deer, duck and quail in Victoria: results from surveys of Victorian game licence holders in 2012. Arthur Rylah Institute for Environmental Research Technical Report Series No. 239. Heidelberg: Department of Sustainability and Environment. 28 p.

[58] Moloney PD, Turnbull JD (2013) Estimates of harvest for deer, duck and quail in Victoria: results from surveys of Victorian game licence holders in 2013. Arthur Rylah Institute for Environmental Research Technical Report Series No. 251. Heidelberg: Department of Environment and Primary Industries. 29 p.

[59] Department of Primary Industries (2013) Victorian hunting guide 2013. Melbourne: Department of Primary Industries. 40 p.

[60] DeVault TL, Rhodes OE, Shivik JA (2003) Scavenging by vertebrates: behavioural, ecological, and evolutionary perspectives on an important energy transfer pathway in terrestrial ecosystems. Oikos 102: 225–234.

[61] Bridges AS, Noss AJ (2011) Behavior and activity patterns. In: O’Connell AF, Nichols JD, Karanth KU, editors. Camera traps in animal ecology: methods and analyses. Berlin: Springer. 57–69.

[62] Forsyth DM, Davis NE (2011) Diets of non-native Sambar Deer in Australia estimated by macroscopic versus microhistological rumen analysis. J Wildl Manage 75: 1488–1497.

[63] Robley A, Gormley A, Forsyth DM, Wilton AN, Stephens D (2010) Movements and habitat selection by wild dogs in eastern Victoria. Aust Mamm 32: 23–32.

[64] Green GI, Mattson DJ, Peek JM (1997) Spring feeding on ungulate carcasses by grizzly bears in Yellowstone National Park. J Wildl Manage 61: 1040–1055.

[65] Henry JD (1977) The use of urine marking in the scavenging behaviour of the red fox (*Vulpes vulpes*). Behaviour 61: 82–105. 869875 10.1163/156853977x00496

[66] Allison PD (1982) Discrete-time methods for the analysis of event histories. Sociol Methodol 13: 61–98.

[67] Congdon PD (2010) Applied Bayesian hierarchical methods. Boca Raton: Chapman and Hall/CRC. 590 p.

[68] R Development Core Team (2013) R: A language and environment for statistical computing. Vienna: R Foundation for Statistical Computing.

[69] Plummer M (2003) JAGS: A program for analysis of Bayesian graphical models using Gibbs sampling. Proceedings of the 3rd International Workshop on Distributed Statistical Computing (DSC 2003), March 20−22, Vienna, Austria.

[70] Su Y-S, Yajima M (2012) R2jags: A package for running JAGS from R. R package version 0.03–08. http://www.CRAN.R-project.org/package=R2jags. Accessed 12 August 2013.

[71] Gelman AB, Carlin JS, Stern HS, Rubin DB (2004) Bayesian data analysis. Second edition. Boca Raton: Chapman and Hall/CRC.

[72] Welton NJ, Ades AE (2005) Estimation of Markov chain transition probabilities and rates from fully and partially observed data: Uncertainty propagation, evidence synthesis, and model calibration. Med Decis Making 25: 633–645. 16282214 10.1177/0272989X05282637

[73] Lunn D, Jackson C, Thomas A, Best N, Spiegelhalter D (2012) The BUGS book: A practical introduction to Bayesian analysis. Boca Raton: Chapman and Hall/CRC. 399 p.

[74] Wood SN (2011) Fast stable restricted maximum likelihood and marginal likelihood estimation of semiparametric generalized linear models. J R Stat Soc Series B Stat Methodol 73: 3–36.

[75] Coe M (1978) The decomposition of elephant carcasses in the Tsavo (East) National Park, Kenya. J Arid Environ 1: 71–86.

[76] Claridge AW, Mills DJ, Hunt R, Jenkins DJ, Bean J (2009) Satellite tracking of wild dogs in south-eastern mainland Australian forests: implications for management of a problematic top-order carnivore. For Ecol Manage 258: 814–822.

[77] Harden RH (1985) The ecology of the dingo in northeastern New South Wales 1. Movements and home range. Aust Wildl Res 12: 25–37.

[78] Doncaster CP, Macdonald DW (1997) Activity patterns and interactions of red foxes (*Vulpes vulpes*) in Oxford city. J Zool 241: 73–87.

[79] Galloway A, Birkby WH, Jones AE, Henry TE, Parks BO (1989) Decay rates of human remains in an arid environment. J Forensic Sci 34: 607–616. 2738563

[80] O’Connell AF, Nichols JD, Karanth KU (2011) Camera traps in animal ecology: methods and analyses. Berlin: Springer. 280 p.

[81] Brawata RL, Neeman T (2011) Is water the key? Dingo management, intraguild interactions and predator distribution around water points in arid Australia. Wildl Res 38: 426–436.

[82] Lazenby BT, Dickman CR (2013) Patterns of detection and capture are associated with cohabiting predators and prey. PLoS ONE 8(4): e59846. 23565172 10.1371/journal.pone.0059846 PMC3614977

[83] Glen AS, Dickman CR (2005) Complex interactions among mammalian carnivores in Australia, and their implications for wildlife management. Biol Rev Camb Philos Soc 80: 387–401. 16094805 10.1017/s1464793105006718

[84] Glen AS, Dickman CR, Soulé ME, Mackey BG (2007) Evaluating the role of the dingo as a trophic regulator in Australian ecosystems. Austral Ecol 32: 492–501.

[85] Newsome AE, Corbett LK, Catling PC, Burt RJ (1983) The feeding ecology of the dingo I. Stomach contents from trapping in south-eastern Australia, and the non-target wildlife also caught in dingo traps. Aust Wildl Res 10: 477–486.

[86] Newsome AE, Catling PC, Corbett LK (1983) The feeding ecology of the dingo II. Dietary and numerical relationships with fluctuating prey populations in south-eastern Australia. Aust J Ecol 8: 345–366.

[87] Robertshaw JD, Harden RH (1986) The ecology of the dingo in north-eastern New South Wales. IV. Prey selection by dingoes, and its effect on the major prey species, the swamp wallaby, *Wallabia bicolor* (Desmarest). Aust Wildl Res 13: 141–163.

[88] Towerton AL, Penman TD, Kavanagh RP, Dickman CR (2011) Detecting pest and prey responses to fox control across the landscape using remote cameras. Wildl Res 38: 208–220.

