# Supplementary material for: How Does a Carnivore Guild Utilise a Substantial but Unpredictable Anthropogenic Food Source? Scavenging on Hunter-Shot Ungulate Carcasses by Wild Dogs/Dingoes, Red Foxes and Feral Cats in South-Eastern Australia Revealed by Camera Traps
Source: PLoS One. 2014 Jun 11;9(6):e97937. doi: 10.1371/journal.pone.0097937 (PMC4053338; doi:10.1371/journal.pone.0097937)
Supplement: Table S1 — Prior distributions for the parameters of the model of time to first carcass visit. (DOC) [file pone.0097937.s001.doc]

**Table S1.** Prior distributions for the parameters of the model of time to first carcass visit.

| Parameter | Prior |
| --- | --- |
| α_1_ (intercept for spring) | Normal(0, 1000) |
| α_2_ (intercept for winter) | Normal(0, 1000) |
| β (distance to fence) | Normal(0, 1000) |
| *k* (shape parameter) | Gamma(1, 0.1) |
| σ_transect_ | Uniform(0, 100) |
